# Are food allergic consumers ready for informative precautionary allergen labelling?

**DOI:** 10.1186/s13223-017-0214-9

**Published:** 2017-10-05

**Authors:** Giovanni A. Zurzolo, Rachel L. Peters, Jennifer J. Koplin, Maximilian de Courten, Michael L. Mathai, Katrina J. Allen

**Affiliations:** 1 Centre for Chronic Disease, College of Health and Biomedicine, Victoria University, Melbourne, Australia; 2 Centre for Food & Allergy Research, Murdoch Childrens Research Institute, Melbourne, Australia; 3 Department of Paediatrics, University of Melbourne, Melbourne, Australia; 4 Department of Allergy and Immunology, Royal Children’s Hospital, Parkville, Melbourne, Australia; 5 Institute of Inflammation and Repair, University of Manchester, Manchester, UK

**Keywords:** Precautionary allergen labelling, Voluntary Incidental Trace Allergen Labelling, Food allergy

## Abstract

Precautionary allergen labelling (PAL) has resulted in consumer confusion. Previous research has shown that interpretive labels (using graphics, symbols, or colours) are better understood than the traditional forms of labels. In this study, we aimed to understand if consumers would use interpretive labels (symbol, mobile phone application and a toll-free number) with or without medical advice that was advocated by the food industry rather than the normal PAL. This is relevant information for industry and clinicians as it provides an insight into the food allergic perception regarding PAL.

## Findings

Precautionary allergen labelling (PAL) has been in place since its voluntary establishment in approximately 2003. Recently several studies have shown that the food industry continual use of PAL is resulting in consumer confusion, reduced quality of life and increased risk-taking since consumers often ignore PAL. Previous research has shown that interpretive labels (using graphics, symbols, or colours) are better understood than the traditional forms of labels these labels may help to reduce the current confusion surrounding PAL [1–3].

In 2007, the Voluntary Incidental Trace Allergen Labelling (VITAL^®^), was developed by the Australian manufacturing industry and is currently managed by the Allergen Bureau. The VITAL^®^ procedure encourages manufacturers to undertake a more intensive investigation into the possible presence of allergens before a product release to consumers. A major limitation of the VITAL^®^ process is that no information is provided to the consumer alerting them that the product in question has undergone a specialised risk assessment and is therefore safe to consume [4]. We have previously highlighted this limitation to industry but labelling to indicate a product has been VITAL^®^ assessed has not being activated.

Food education allows individuals to build knowledge and values, reframe their food practices, and develop strategies for a healthy and safe diet.

In this study, we aimed to understand if consumers would use a symbol which was advocated by the Allergen Bureau on food products that had undergone the VITAL^®^ process and represented a very low level of cross contact. We also examined if consumers would use a mobile phone application or a toll-free number to access information when buying food products.

The methods of this study are described elsewhere [5] but in brief, 535 participants were recruited from the Department of Allergy and Immunology at the Royal Children’s Hospital, Melbourne. 497 children (93%) agreed to participate. Food allergy had been medically diagnosed in 293 (59%) Of the 293 children with food allergy, 246 (84%) had sufficient information provided to allow past reactions to be classified as either a past history of anaphylaxis (113 children) or a past history of mild to moderate IgE mediated reactions (133 children).

We presented to the participants three different methods of labelling. The three questions were:

1. Participants were asked to consider if the “may be present” symbol was used to represent a LOW level of cross contamination, (an amount that is so low that will be unlikely to cause a severe allergic reaction) would they find this statement useful and consume foods with this statement, or consume foods with this statement only if your doctor or allergy specialist said it was safe to do so. (The participants were given no information regarding VITAL^®^ or its processes).
2. If there was an independent toll free number listed on all food products would they call to gain more information regarding the products.
3. If there was a mobile phone application developed in which they could scan the barcode of a food product and instantly receive more information regarding the ingredients.

**Fig. 1:**
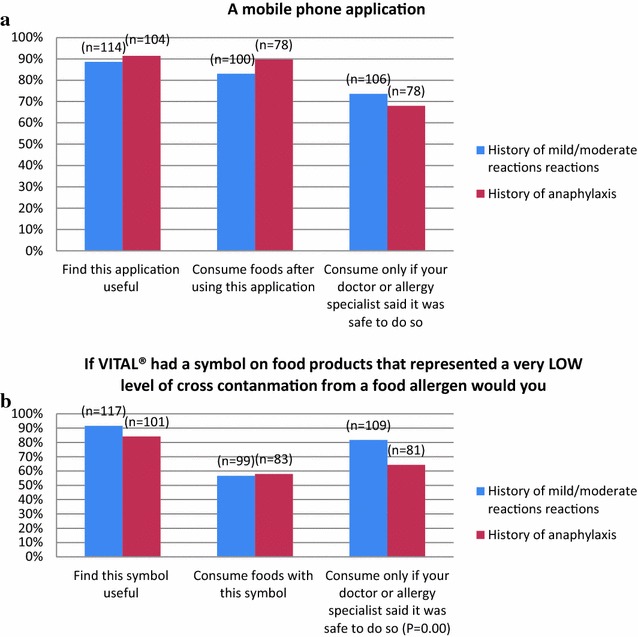
**a** Food allergic participants were asked to consider the above symbol been placed on food products and if this simple would be useful, would they consume foods with this symbol or would they consume foods with this symbol if advised by their healthcare provider? **b** Food allergic participants were asked to consider if there was a mobile phone application in which they could scan the barcode of a food product and instantly receive more information regarding the ingredients

Responders that reported on the usefulness of the proposed VITAL^®^ symbol, 91% (n = 117) of participants that had mild to moderate reactions and 84% (n = 101) of participants that had a history of anaphylaxis reported that they would find this symbol useful. A lesser portion of responders reported that they would consume foods with these symbol 56% (n = 99) and 57% (n = 83) respectively. However this increased when asked if they would consume foods with this symbol if advised by their healthcare provider that it was safe to do so 81% (n = 109) and 64% (n = 81) respectively (Fig. 1a).

Responders that reported on the usefulness of a mobile phone application, 88% (n = 114) of participants with a history of mild to moderate reactions and 91% (n = 104) with a history of anaphylaxis reported that they would find this application useful. This proportion remained similar when asked if they would consume foods after using this application but slightly dropped when asked if they required their healthcare provider to inform them that it was safe to do so 73% (n = 106) and 67% (n = 78) respectively (Fig. 1b).

**Fig. 2:**
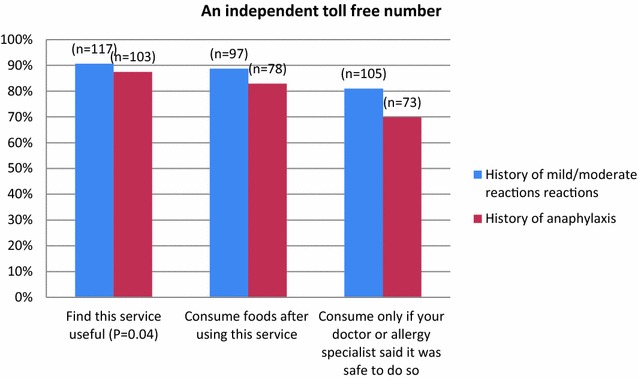
Food allergic participants were asked to consider if there was an independent toll free number listed on all food products that they could call to gain more information regarding the product’s ingredients

Responders that reported on the benefit of an independent toll-free number, 90% (n = 117) of participants with a history of mild to moderate reactions and 87% (n = 103) with a history of anaphylaxis reported that they would find this service useful. This proportion remain similar when asked if participants would consume foods after using this service and slightly dropped when asked if they would only consume foods from this service if their healthcare provider told them it was safe to do so 80% (n = 105) and 70% (n = 73) respectively (Fig. 2).

In this current study we presented to participants three different methods of information delivery for food labelling. The results show that the majority of responders would find all three very useful if they were placed on package goods. For the mobile phone application and the toll-free number, the majority of responders would consume food products without seeking endorsement from their healthcare provider. This may be due to the fact that participants felt receiving live information via a mobile phone application or a toll-free number was sufficient enough information for them to support their decision to eat the product. However further studies will be required to examine the specific nature of the information given via mobile phone application or toll-free numbers in order to assess how much information is sufficient to bring about change in their behaviour before changes to policy can be recommended. In regards to the VITAL^®^ symbol a greater portion of responders (81% mild-to-moderate reactions and 64% history of anaphylaxis p = 0.00), would only consume foods with this symbol if their healthcare provider instructed them that it was safe to do so. A possible explanation to this may be the uncertainty and legitimacy around the VITAL^®^ statement.

The key strengths of our study are the response rate of 93%, and that participants received no education in relation to the VITAL^®^ process, therefore it is unlikely that the participant bias in favour of VITAL^®^ was present. A limitation to this study is that the results depend on what an individual states that they would or would not do and does not actually quantify whether this would correlate with action. Further studies would be required in order to examine this question. Another possible limitation is that we relied on parents’ self-report that their child had medically diagnosed food allergy and a past history of anaphylaxis. However, we believe this is appropriate for this type of study as parents’ perceptions and attitudes are likely to drive their choices when making decisions on behalf of their children.

Souza et al. analyse 702 individuals to understand the effectiveness of an educational intervention regarding food labelling as a tool to promote public health. Participants were asked to complete a questionnaire regarding food labelling. Thirty days after the first initial questionnaire participants were asked to complete the same questionnaire but this time the participants were provided with a folder of educational material to promote the understanding of food labels. The results from the first questionnaire showed that, 55.8% of the respondents reported consulting information provided on packaged foods, however 30 days later 72.0% of respondents reported consulting this information (p < 0.001) [6].

Currently there is no education or information on food products that have been through the VITAL^®^ process that alerts the consumer regarding this process.

There is substantial evidence indicating that interpretive labels (using graphics, symbols, or colours) are better understood than the traditional numerical nutrition labels [3].

Our research shows that consumers would benefit from utilising any of the three different methods of labelling that were examined in this study and if these methods of labelling were delivered to consumers with appropriate education regarding the VITAL^®^ process consumers would be able to consume foods without the added stress, anxiety and uncertainty that currently exist around packaged goods.

## Abbreviations

PAL: precautionary allergen labelling
VITAL: Voluntary Incidental Trace Allergen Labelling
